# Supplementary material for: Prevalence of ocular trauma and barriers to use of personal protective devices among welders in Hetauda, Nepal
Source: PLoS One. 2025 Dec 30;20(12):e0329050. doi: 10.1371/journal.pone.0329050 (PMC12753074; doi:10.1371/journal.pone.0329050)
Supplement: Attachment — Submitted filename: Response to reviewers.docx [file pone.0329050.s003.docx]

Dear Editor and Reviewers,

Manuscript title: Prevalence of Ocular Trauma and Barriers to Use of Personal Protective Devices Among Welders in Hetauda, Nepal

Authors: Sunil Thakali, Mohini Shrestha, Aleena Gauchan, Dikshya Bista, Hom Bahadur Gurung

We sincerely thank the Academic Editor and all reviewers for their thorough evaluation and constructive comments. Each suggestion has been carefully considered, and the manuscript has been extensively revised to improve clarity, scientific accuracy, and adherence to PLOS ONE formatting requirements. All changes are highlighted in the file titled “Revised Manuscript with Track Changes.”

A detailed, point-by-point response follows.

**Reviewer #1**

Comment 1: The use of “PPE” abbreviation is inappropriate; please spell out in the first instance.

Response: Revised throughout. “Personal protective equipment (PPE)” is now written in full at first mention in both the Abstract and Introduction.

Comment 2: Strengthen the Introduction by reviewing similar international articles.

Response: Added recent global references, including Yan et al. (2022) and Lombardi et al. (2005), as well as additional studies from 2019–2024, to provide stronger international context.

Comment 3: Provide sample-size calculation.

Response: A complete description of the sample-size calculation using the single-proportion formula has been added under Study Design and Sample Size.

Comment 4: Clarify inclusion criteria—were all participants examined?

Response: Stated explicitly that all registered welders, regardless of ocular complaints, underwent complete ocular examinations.

Comment 5: Cite the Ghana study and justify questionnaire modifications.

Response: The source (Tetteh et al., 2020) has been cited, and modifications (local language, equipment terminology, occupational context) are explained with justification.

Comment 6: Define ocular trauma.

Response: Added explicit operational definition encompassing self-reported or clinically confirmed mechanical, thermal, or chemical injuries.

Comment 7: Table 3 layout “Own PPE” should appear under “PPE ownership and use.”

Response: Table 3 reorganized accordingly.

Comment 8: “Trained” and “Untrained” should be adjacent.

Response: Corrected in the revised table.

Comment 9: Compare Nepali results with global data.

Response: Added comparative discussion referencing Ghana, Nigeria, and U.S. statistics to contextualize Nepal’s prevalence.

Comment 10: Male predominance—please elaborate.

Response: Discussion expanded to note welding’s male-dominated workforce in Nepal and implications for sex-based analyses.

Comment 11: Expand on cost of equipment and its effect on PPE adherence.

Response: Added explanation linking affordability and availability to inconsistent PPE use; proposed subsidized or employer-provided devices.

Comment 12: Add limitation about need for long-term studies.

Response: Included in the Limitations section.

Comment 13: Many cited papers are old.

Response: Updated multiple citations with recent studies (2019–2024).

Comment 14: Figures are low quality.

Response: All figures regenerated at ≥300 dpi TIFF resolution per PLOS ONE requirement

**Reviewer #2**

Comment 1: Abstract should follow structured format.

Response: Abstract rewritten into four sections—Introduction, Methods, Results, and Conclusions.

Comment 2: Use PubMed MeSH terms for keywords.

Response: Keywords revised to standardized MeSH terms: “Occupational Eye Injuries,” “Welding,” “Personal Protective Equipment,” “Occupational Health,” “Workplace Safety.”

Comment 3: Begin introduction with international data.

Response: Added global statistics on occupational eye injuries at the start of the Introduction.

Comment 4: Discuss health and economic burdens.

Response: Included global and national cost implications of ocular injuries.

Comment 5: Clarify research gap and study significance.

Response: Final paragraph of Introduction rewritten to highlight limited Nepal-specific evidence and study relevance

Comment 6: Ethical considerations should be stated.

Response: Ethical clearance (Ref No. 07/2024, Tilganga Institute of Ophthalmology) and consent process detailed in Ethical Clearance subsection.

Comment 7: Clarify sampling method.

Response: Described as convenience sampling among welders registered with the Grill and Steel Fabrication Association of Makwanpur.

Comment 8: Include table summarizing questionnaire components.

Response: Added new Table 1: Questionnaire Components summarizing sections and number of questions.

Comment 9: Specify data-analysis software and tests.

Response: Added that analyses were performed in R (v 2025.05.1) using logistic regression with significance set at p < 0.05.

Comment 10: Expand the Conclusions section to interpret implications.

Response: Conclusions rewritten to emphasize public-health and occupational-safety implications rather than restating results.

**Reviewer #3**

Comment 1: Indicate total number of welders in the association and response rate.

Response: Added estimated total (≈ 600 welders) and response rate (18.5%) to Study Design.

Comment 2: Include years of experience and analyze association.

We thank the reviewer for this valuable suggestion. Unfortunately, data on years of experience were not collected in our study proforma, so we are unable to analyze its association with ocular trauma among welders in this dataset. We acknowledge that years of experience is an important factor and recommend that future studies in Nepal include this variable to better understand its role in occupational eye injuries.

Comment 3: Report ocular-trauma proportion among female welders.

Response: Added finding that 2 of 8 female participants (25%) reported ocular trauma.

Comment 4: Recall bias limits generalizability.

Response: Expanded discussion of recall bias and acknowledged its potential effect on self-reported injuries.

**Reviewer #4**

Comment: Manuscript lacks novelty.

Response: We thank the reviewer for their comment and appreciate the opportunity to clarify the contribution of our study. While previous studies have examined ocular trauma among welders globally, there is a scarcity of data specific to Nepal, particularly regarding both prevalence and associated risk factors in our local industrial context. This is the first investigation from Hetauda, Nepal, quantifying local prevalence and identifying context-specific barriers to protective-device use—crucial baseline data for regional occupational-health policy.
