# Supplementary material for: Prevalence of ocular trauma and barriers to use of personal protective devices among welders in Hetauda, Nepal
Source: PLoS One. 2025 Dec 30;20(12):e0329050. doi: 10.1371/journal.pone.0329050 (PMC12753074; doi:10.1371/journal.pone.0329050)
Supplement: Attachment — Submitted filename: Response_to_reviewers_auresp_2.docx [file pone.0329050.s004.docx]

Dear Editor and Reviewers,

Manuscript title: Prevalence of Ocular Trauma and Barriers to Use of Personal Protective Devices Among Welders in Hetauda, Nepal

Authors: Sunil Thakali, Mohini Shrestha, Aleena Gauchan, Dikshya Bista, Hom Bahadur Gurung

We sincerely thank the Academic Editor and both reviewers for their valuable time, constructive feedback, and insightful comments that have helped us improve the quality and clarity of our manuscript. We have carefully revised the paper in response to each point raised. A detailed, point-by-point response follows.

**Response to Reviewer #2**

Comment 1: While thanking you for the corrections made in this article regarding the questionnaire, due to the changes in the questions, the validity of the questionnaire has not been explained. What is meant by the changes is unclear. Due to the changes in the references, the changes are not clear.

Response: Thank you for your valuable comment. The questionnaire used in this study was adapted from a validated study conducted among welders in Accra, Ghana [1]. Minor modifications were made to ensure cultural and occupational relevance for Nepal, including adjustments to occupational history questions to reflect local work practices, inclusion of Nepali terms for personal protective equipment, and adaptation of items to match the types of welding equipment commonly used in Hetauda workshops. These modifications were reviewed by occupational health and ophthalmology experts to maintain content validity. Additionally, the adapted questionnaire was pilot-tested among a small group of welders (n = 10) to ensure clarity, comprehension, and contextual appropriateness before full-scale data collection.

**Response to Reviewer #3**

Comment 1: Please, kindly mention the number of all the registered welders that were initially invited for the visual screening out of which 111 welders consented to present themselves for the study.

Response: All welders invited (n = 111) consented and participated in the study, giving a response rate of 100%. This has been clearly mentioned in the Results section.

Comment 2: Kindly define Frequent Safe Training and Sometimes Safe Training at work mentioned in Table 4.

Response: These terms have now been defined in a footnote of Table 4 as follows:

- Frequent Safe Training: Conducted regularly (at least once per year) with structured safety instructions on PPE use.
- Sometimes Safe Training: Conducted irregularly or informally, without consistent follow-up or formal structure.

Comment 3: Was fundoscopy done for any of the patients? No posterior segment ocular morbidity was mentioned. Was intraocular pressure done for any of the patients?

Response: Yes, fundoscopy was performed using a 90D lens under slit-lamp examination; no posterior segment abnormalities were found. Intraocular pressure (IOP) was measured for all participants using a non-contact tonometer (iCARE). These details have been added to the Methods section under Ophthalmic Examination.

Comment 4: Please include the number of years each participant had been involved in welding.

Response: We acknowledge that the number of years of welding experience was not recorded in our study proforma. This has now been added as a limitation in the Discussion section, and we recommend including it in future research to better assess its relationship with ocular morbidity.

Comment 5: Maculopathy is an important ocular morbidity in welders.

Response: Thank you for the observation. Fundoscopic examination did not reveal any cases of maculopathy among participants. This information has been clarified in the Results section.
